# Green Membrane Technologies: Advancements in Materials and Energy Efficiency for Water Treatment

**DOI:** 10.3390/membranes16050168

**Published:** 2026-05-04

**Authors:** Jehad A. Kharraz

**Affiliations:** 1 Department of Chemical and Petroleum Engineering, Khalifa University of Science and Technology, Abu Dhabi P.O. Box 127788, United Arab Emirates; jehad.kharraz@ku.ac.ae; 2 Center for Membranes and Advanced Water Technology (CMAT), Khalifa University of Science and Technology, Abu Dhabi P.O. Box 127788, United Arab Emirates

## 1. Introduction

The growing global water crisis continues to place unprecedented pressure on water treatment technologies to deliver higher efficiency, lower energy consumption, and reduced environmental impacts. Membrane processes—already central to desalination and wastewater reclamation—are now being challenged to evolve beyond performance metrics alone and embrace sustainability at the material, process, and systems levels. This Special Issue “Green Membrane Technologies: Advancements in Materials and Energy Efficiency for Water Treatment” was conceived with this broader transition in mind.

Over the past decade, membrane science has made remarkable strides in permeability enhancement, selectivity control, and fouling mitigation. Yet several gaps remain. The environmental footprint of membrane fabrication, long-term energy demand in large-scale desalination systems, management of complex industrial effluents, and integration with renewable energy infrastructures continue to limit the realization of truly sustainable water treatment. Furthermore, circular-economy principles, while frequently discussed, have not been consistently embedded within membrane system design and operation.

Globally, desalination capacity now exceeds 140 million m^3^/day, with reverse osmosis accounting for approximately 70–75% of the installed capacity [1]. While RO has dramatically reduced specific energy consumption compared to thermal desalination [2], brine production remains a major environmental concern, with concentrated reject streams often exceeding 120 million m^3^/day [1]. The environmental burden of brine disposal, chemical cleaning, and membrane replacement complicates the narrative that pressure-driven membranes are inherently “green.”

At the material level, conventional polymeric membranes rely heavily on solvent-intensive phase inversion processes, frequently using toxic solvents such as NMP or DMF [3]. Ceramic membranes offer durability and chemical stability but are typically sintered at temperatures above 1000 °C, raising embodied-energy concerns. At the operational level, fouling remains the dominant contributor to energy escalation, increased cleaning frequency, and shortened membrane lifespans [4]. At the system level, high-recovery concepts such as Zero Liquid Discharge (ZLD) promise wastewater elimination but often at the cost of elevated thermal energy demand [5].

These realities signal that sustainable membrane engineering must move beyond incremental flux enhancement. It must address lifecycle energy, material circularity, fouling-resilient design, and integration with resource recovery frameworks. The contributions assembled in this Special Issue collectively reflect this multidimensional transition.

## 2. Contributions to This Special Issue

The study by Bahrouni et al. (2025) (Contribution 1) advances the sustainability agenda at the material level through the development of clay-based ceramic membranes incorporating almond shell waste and lime as additives. By reducing sintering temperatures to 900–1000 °C, a span below conventional ceramic-processing ranges, the authors demonstrated that mechanical robustness and filtration performance can be preserved while lowering thermal energy demand. The membranes exhibited a porosity of between 26 and 30%, average pore sizes of around 42–44 nm, and pure-water permeability of up to 68 L·m^−2^·h^−1^·bar^−1^. Textile dye removal efficiencies reached 92% for indigo blue, with more than 90% permeability recovery after repeated cleaning cycles. This work is not only significant because of its performance metrics but because it embeds agricultural waste valorization directly into membrane fabrication, aligning material design with circular-economy principles and reduced embodied energy.

At the process-design level, Jeon et al. (2025) (Contribution 2) re-examined forward osmosis (FO) module configuration to address energy efficiency constraints inherent in serial draw-solution dilution. Their proposed draw-solution split distribution (DSSD) configuration maintained osmotic driving force across modules, achieving enrichment ratios of 12.5 at 0.137 kWh/m^3^—outperforming conventional serial FO (2.5 at 0.151 kWh/m^3^) and demonstrating lower energy consumption compared to reverse osmosis systems operating at similar concentration ratios. Rather than modifying membrane chemistry, the authors underscore the importance of hydraulic architecture and system-level optimization in achieving energy-efficient dewatering. Such contributions reinforce the view that sustainable membrane engineering must extend beyond material innovation and move toward module design and process integration.

Shi et al. (2025) (Contribution 3) explored the convergence of membrane separation and electrochemical functionality through electro-conductive PVDF membranes fabricated via in situ oxidative polymerization of polypyrrole. Under low applied voltages (0.5–2.0 V), fouling rates during filtration of model organic foulants were significantly reduced, with humic acid fouling decreasing by over 70% under optimized conditions. The membrane achieved electrical resistance as low as 93 Ω/sq while maintaining hydrophilicity. Electrified membrane systems represent a promising direction for chemical-free fouling mitigation and enhanced process stability. However, they also require careful assessment of net energy balance and long-term stability under continuous operation. This work illustrates how multifunctionality—rather than single-property optimization—may define the next generation of green membrane technologies.

Moving beyond liquid streams, Collivignarelli et al. (2025) (Contribution 4) addressed sludge management through the integration of a thermophilic aerobic membrane reactor (TAMR) into a wastewater treatment plant. The system achieved an up to 90% reduction in volatile solids and reduced sludge output to approximately 10% of conventional levels. Given that sludge handling can constitute up to 50% of operational costs in wastewater treatment facilities, such reductions carry both economic and environmental implications. Importantly, the STAR configuration demonstrated compatibility between thermophilic treatment residues and conventional activated sludge systems, enabling nutrient recirculation and superior circularity. This contribution expands the scope of membrane sustainability beyond filtration performance and toward integrated plant-wide resource recovery.

Finally, the review by Panagopoulos and Michailidis (2025) (Contribution 5) contextualizes membrane technologies within ZLD and MLD frameworks. The authors highlight that while RO is dominant in terms of desalination capacity, high-salinity brine management remains unresolved, driving interest in hybrid membrane configurations incorporating RO, FO, membrane distillation (MD), and crystallization stages. Their synthesis emphasizes that achieving near-complete water recovery must be balanced against escalating energy demand and scaling risks. The review situates membrane innovation within global sustainability goals, particularly resource recovery and brine valorization, reinforcing the need for system-level optimization.

The five contributions demonstrate a progression from sustainable material fabrication to energy-optimized module design, multifunctional electrified membranes, sludge minimization, and finally strategic ZLD integration. The thematic coherence of this Special Issue lies not in a single technological pathway but in its shared commitment to embedding sustainability across scales.

## 3. Outlook: Toward Carbon-Aware, Circular, and Intelligent Membrane Systems

The trajectory of green membrane technologies must now be guided by quantitative sustainability metrics rather than qualitative claims. Lifecycle carbon accounting of membrane fabrication, solvent usage, sintering energy, operational electricity demand, and cleaning frequency should become standard reporting practice. Without such transparency, claims of “low-energy” or “green” performance remain incomplete.

Future research must also reconcile electrification and the integration of renewable energy with process stability. Electrified membranes and hybrid systems introduce new control variables that require advanced monitoring and optimization frameworks. In parallel, artificial intelligence and data-driven modeling offer significant opportunities for predictive fouling control and dynamic process management, reducing unnecessary chemical cleaning and energy expenditure.

Brine management and sludge minimization must evolve from waste containment into resource extraction. Selective ion recovery, nutrient reclamation, and integration with industrial symbiosis platforms may define the economic feasibility of future ZLD systems. Importantly, pilot-scale validation and long-term durability studies will be essential to translate laboratory innovations into operational reality.

This Special Issue reflects a field in transition, moving from performance-driven separation science toward carbon-aware, circular, and system-integrated engineering. The convergence of material innovation, energy-efficient process design, electrochemical functionality, and resource recovery strategies signals that green membrane technologies are no longer peripheral research topics but rather central to the future of sustainable water treatment.

It is hoped that the contributions presented here will stimulate further interdisciplinary collaboration and accelerate the evolution of membrane systems that are not only efficient but also genuinely sustainable.
